# Reticulocyte hemoglobin content: a new frontier in iron deficiency diagnostics for major surgical patients

**DOI:** 10.1186/s12871-025-02905-6

**Published:** 2025-01-25

**Authors:** Suma Choorapoikayil, Mischa J. Kotlyar, Lisa Kawohl, Paul P. Pratz, Denana Mehic, Peter Kranke, Florian Rumpf, Lea V. Blum, Jan A. Kloka, Kai Zacharowski, Vanessa Neef, Patrick Meybohm

**Affiliations:** 1 Goethe University Frankfurt, University Hospital Frankfurt, Department of Anaesthesiology, Intensive Care and Pain Therapy, Frankfurt, Germany; 2 University Hospital Würzburg, Department of Anaesthesiology, Intensive Care, Emergency and Pain Medicine, Würzburg, Germany

**Keywords:** Hemoglobin equivalent, Ret-He, Surgery, Ret-Hb, Anemia screening

## Abstract

**Background:**

Iron deficiency (ID) is the most common nutritional deficiency among patients undergoing major surgery. Treatment of ID is straightforward, however implementing a comprehensive anemia management strategy within clinical routines is complex. Recently, reticulocyte hemoglobin content (Ret-He) has been evaluated as an early marker for ID diagnosis.

**Method:**

In this retrospective study, 2,966 major surgical patients from two University Hospitals were screened for the presence of ID and the significance of Ret-He in diagnosis ID was evaluated in both non-anemic and anemic patients. According to hemoglobin, ferritin, and transferrin saturation concentrations patients were assigned to a Control group (no anemia, no ID), ID group (no anemia, ID), IDA group (anemia, ID) or Others group (anemia, no ID).

**Results:**

In total, 2,760 patients were included in analysis: Control (*n* = 1500; 54.2%), IDA (*n* = 412; 14.9%), ID (*n* = 487; 17.6%), and Others (*n* = 370; 13.4%). Ret-He was significantly decreased in the IDA group compared to ID, Control and Others, respectively (*p* < 0.001). The ROC curve analysis revealed an AUC of 0.842 (95% CI (0.82–0.87)) at Ret-He cutoff 33.5 pg, by which IDA was discriminated with 69.7% (95% CI (65.3–74.0%)) sensitivity and 85.7% (95% CI (82.3-86.1%)) specificity. Of the 370 patients with anemia of unknown cause (Others group) 131 had Ret-He levels < 33.5 pg. In these patients, the median values for ferritin was 492.0 ng/ml (333.5; 818.5 ng/ml) and transferrin saturation 11.9% (18.0; 23.3%). Logistic regression identified significant predictors of ID, with each decrease in Hb and Ret-He associated with a 19.4% (OR = 0.806; *p* < 0.001) and 26% (OR = 0.740; *p* < 0.001) increase in the odds of ID, respectively.

**Conclusion:**

This study highlights the potential of Ret-He as a promising alternative marker for diagnosing ID in patients undergoing major surgery, particularly in cases of elevated ferritin levels or non-anemic patients. Ret-He may serve as a valuable tool to prioritize patients for further iron status testing, especially when preoperative time is limited.

**Supplementary Information:**

The online version contains supplementary material available at 10.1186/s12871-025-02905-6.

## Introduction

Iron deficiency (ID) is the most common nutritional deficiency in the population and the leading cause of anemia with a prevalence of 23 to 33% [1]. In clinical setting, preoperative anemia is associated with an increased risk for perioperative complications and mortality. Triphaus et al. showed that every second major surgical patients with a hemoglobin (Hb) level below 10 g/dl had ID [2]. Both oral and intravenous administration of iron are effective and low-cost measures to treat anemia [3]. However, it is important to recognize that treatment requires time to show its full effect. Oral iron typically takes three to six months, while intravenous iron can take at least seven days to improve iron levels and erythrpoiesis. Therefore, early detection of ID is crucial to ensure timely and effective management.

The diagnosis of ID is challenging. The cutoff values and measurements to diagnose ID have been rigorously debated in the last decades. The World Health Organization (WHO) recommends a ferritin cutoff value of < 15 μg/l in healthy individuals and < 70 µg/l in individuals with infection or inflammation for the diagnosis of ID [4]. In the patients with chronic kidney disease or heart failure, ferritin values of up to 300 ng/ml and transferrin saturation below 20% are also considered indicative for ID [5]. However, ferritin is an acute phase protein and often elevated if inflammation is present. Therefore, Muñoz and colleagues recommend a simultaneous measurement of ferritin, transferrin saturation and C-reactive protein (CRP) in surgical patients [5, 6].

The detrimental impact of preoperative anemia has been consistently demonstrated. However, implementing a comprehensive anemia management strategy within clinical routines is complex. The limited time available between preoperative assessment and surgery necessitates a straightforward screening and treatment process to ensure accurate diagnosis and management of ID. Diagnostic accuracy is often challenged by factors such as inflammation or chronic disease, which can obscure true ID. Additionally, the lack of standardized protocols can lead to variability in how ID is screened and managed across different clinical settings. Since performing laboratory analysis for ID is costly, a pre-selection of potentially iron-deficient patients would be beneficial. Resource constraints in some healthcare environments further complicate access to full diagnostic panels, and integrating new diagnostic processes into established clinical workflows can be difficult. Furthermore, patient-specific factors, including comorbidities and nutritional status, add another layer of complexity to the diagnosis and management of ID.

Recently, reticulocyte hemoglobin content (Ret-He) has been evaluated as an early marker for ID diagnosis [7]. Reticulocytes are immature red blood cells that reflect the responsiveness of the bone marrow, and their hemoglobin content correlates with the functional availability of iron for erythropoiesis. In contrast to ferritin, Ret-He is not influenced by inflammation. It is the first peripheral blood count marker being abnormal in the presence of ID. Ret-He has a short life span of 1–2 days and enables an early detection of ID [8]. However, there are currently no standardized thresholds for Ret-He in the diagnosis of ID. This study investigated the significance of Ret-He in diagnosis of ID in major surgical patients.

## Methods

### Study design

This was a retrospective study performed at the University Hospital Frankfurt and University Hospital Würzburg. The study protocol was approved by the ethics committee of the University Hospital Frankfurt (Ref. 318/17) and the requirement for written informed consent by patients was waived. Data were extracted from the anemia outpatient clinic of both hospitals.

### Cutoff values

Anemia was defined according to the WHO, with Hb values of < 12 g/dl for women and < 13 g/dl for men considered anemic [9]. Iron deficiency was defined by serum ferritin < 100 ng/ml or serum ferritin 100–300 ng/ml and transferrin saturation < 20% [5, 6]. Accordingly, patients were assigned to a Control group (no anemia, no ID), ID group (no anemia, ID), IDA group (anemia, ID) or Others group (anemia of unknown cause, no ID).

Reticulocyte hemoglobin content was measured by fluorescence flow cytometry in the reticulocyte channel of the XN-1000 or XN-9000 (Sysmex, Norderstedt, Germany).

### Statistical analysis

Descriptive variables were calculated using medians and interquartile ranges (IQRs, P25%; P75%). Statistical significance was considered with *P* < 0.05. A Kruskal–Wallis test was conducted to assess differences in Ret-He levels across the groups. Post hoc comparisons were performed using Dunn's test with Benjamini–Hochberg correction to control for false discovery rate in multiple comparisons.

Spearman's rank correlation coefficient was used to assess the strength and direction of the monotonic relationship between Ret-He and ferritin and transferrin saturation, respectively.

Receiver Operating Characteristic (ROC) curve analysis was conducted to evaluate the diagnostic performance of Ret-He in identifying ID. The area under the ROC curve (AUC) was calculated to quantify the overall accuracy of Ret-He as a diagnostic marker, with an AUC of 0.5 indicating no diagnostic ability and an AUC of 1.0 indicating perfect discrimination. The Youden Index was used to identify the optimal threshold, which maximizes the sum of sensitivity and specificity by balancing the true positive rate and false positive rate (1-specificity). The 95% confidence interval (CI) was calculated using bootstrapping with the pROC package in R. In addition to ROC analysis, classification performance was further assessed using accuracy, precision, recall (sensitivity), and F1 score. Accuracy measures the proportion of correctly classified cases, while precision (positive predictive value) evaluates the proportion of true positive results among those identified as positive by the model. Recall (sensitivity) reflects the proportion of true positive cases correctly identified, and the F1 score, the harmonic mean of precision and recall, provides a balanced measure of the test's performance, especially in the presence of class imbalance [10]. In addition, a fivefold cross-validation approach was performed. For that, the dataset was randomly partitioned into five equal subsets, with four subsets used for training and one for testing in each iteration. This process is repeated five times, ensuring that each subset is used for testing once, which provides a robust estimate of the model's performance and reduces the risk of overfitting. We calculated the 95% CIs for the AUC, sensitivity, and specificity of the fivefold cross-validation model using the normal approximation formula, CI = mean ± 1.96 × SD / √5. The mean and standard deviation (SD) were computed from the scores across the 5-folds.

A logistic regression analysis was performed to evaluate the association between Hb, Ret-He, CRP, mean corpuscular volume (MCV), mean corpuscular hemoglobin (MCH) and anemia status and the likelihood of ID.

Due to the retrospective design of the study, a power analysis was not performed, and the sample size was determined based on the available data.

All analyses and graphical illustrations were performed using R software (version 3.1–124), and Excel (2016). The following packages were used in R: (psych); (FSA); (lattice); (pROC); (ggplot2); (stats); (caret); (mlogit); (dplyr).

## Results

Between September 2017 and January 2023 2,966 patients undergoing major surgery were screened for the presence and possible causes of anemia in the anemia outpatient clinic at the University Hospital Frankfurt and University Hospital Würzburg. Of the 2,966 patients 2,769 had laboratory values for Hb, ferritin, transferrin saturation, and Ret-He and were included in analysis. Among them 487 (17.6%) patients had ID, 412 (14.9%) patients had IDA and 370 (13.4%) patients had anemia but no ID. The total prevalence of preoperative anemia was 28.2%.

**Fig. 1:**
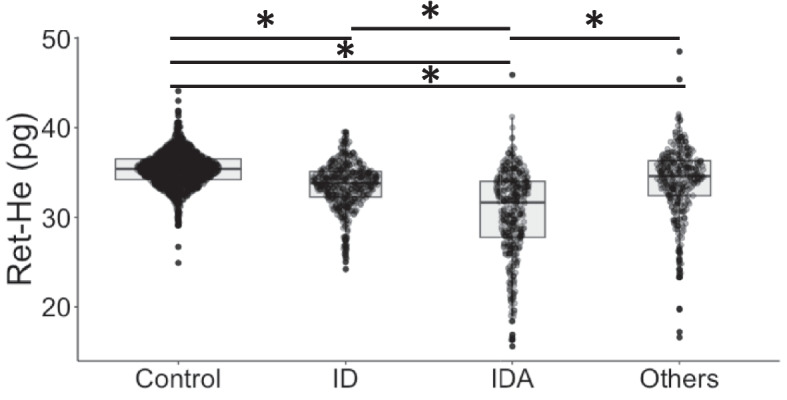
Distribution of Reticulocyte Hemoglobin Content between control, ID, IDA, and Others. ID = Iron deficiency; IDA = Iron deficiency anemia; Ret-He = Reticulocyte Hemoglobin Content; * = significant differences

The proportion of female patients was highest in the ID group with 45.4% compared to the IDA group with 38.3% and to the Others group with 25.1%. Patients of the IDA (70 (61; 78) years) and Others (70 (62; 77)) group were significantly older compared to patients of the Control (66 (57; 73)) and ID (64 (55; 73)) group (*p* < 0.001) (Table 1). Ret-He was significantly decreased in patients of the IDA group compared to ID, Control and Others, respectively (*p* < 0.001) (Table 1, Fig. 1).

**Table 1:** Descriptive statistics

	**Control** **(*n* = 1500)** **54.2%**	**IDA** **(*n* = 412)** **14.9%**	**ID** **(*n* = 487)** **17.6%**	**Others** **(*n* = 370)** **13.4%**
Age (years)	66 (57; 73)	70.0 (61; 78)	64(55; 73)	70 (62; 77)
Gender (female)	360 (24%)	158 (38.3%)	221 (45.4%)	93 (25.1%)
Hb (g/dl)	14.5 (13.7; 15.2)	11.1 (9.9; 11.0)	13.7 (13.1; 14.6)	11.3 (10.1; 12.3)
MCV (fl)	88.2 (85.6; 91.1)	85.2 (79.5; 89.5)	86.7 (84.0; 89.3)	89.7 (85.6; 93.8)
MCH (pg)	30.4 (29.5; 31.6)	28.5 (25.7; 30.1)	29.4 (28.4; 30.6)	30.1 (28.7; 31.9)
Ret-He (pg)	35.4 (34.2; 36.5)	31.7 (27.8; 34.0)	33.8 (32.3; 35.1)	34.6 (32.4; 36.3)
Ferritin (ng/ml)	217.0 (116.0; 361.0)	62.0 (27.0; 130.0)	93.0 (39.0; 169.0)	405.5 (227.0; 692.8)
Transferrin saturation (%)	27.0 (23.5; 33.5)	11.8 (7.8; 17.3)	15.8 (12.8; 18.1)	23.9 (18.3; 31.4)
CRP (mg/dl)	0.2 (0.1; 0.4)	0.5 (0.2; 1.5)	0.4 (0.2; 0.9)	0.7 (0.2; 2.9)

*Hb* hemoglobin, *MCV* mean corpuscular volume, *MCH* mean corpuscular hemoglobin, *Ret-He* Reticulocyte hemoglobin content, *CRP* C-reactive protein, median values are displayed

### Correlation of Ret-He with ferritin and transferrin saturation

The comparison of all patients and the different study groups revealed a weak correlation between Ret-He and ferritin (rho = 0.274) and a moderate correlation between Ret-He and transferrin saturation (rho = 0.543) (Additional Table 1, Additional Fig. 1).

### Receiver-operating characteristic analysis of RET-He in the diagnosis of iron deficiency

**Fig. 2:**
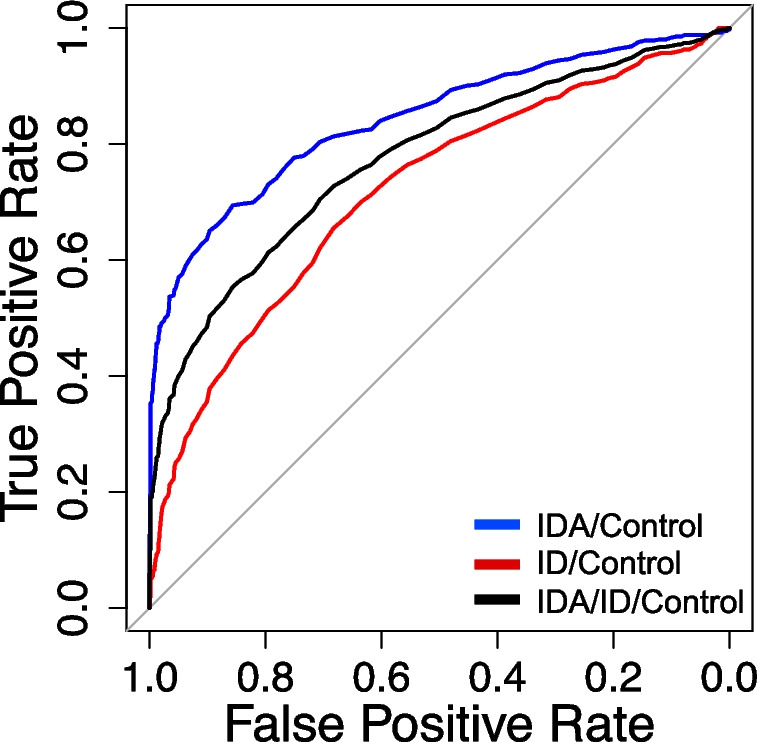
Receiver-operating characteristic analysis of Ret-He in the diagnosis of iron deficiency. ID = Iron deficiency; IDA = Iron deficiency anemia; Ret-He = Reticulocyte Hemoglobin Content

**Fig. 3:**
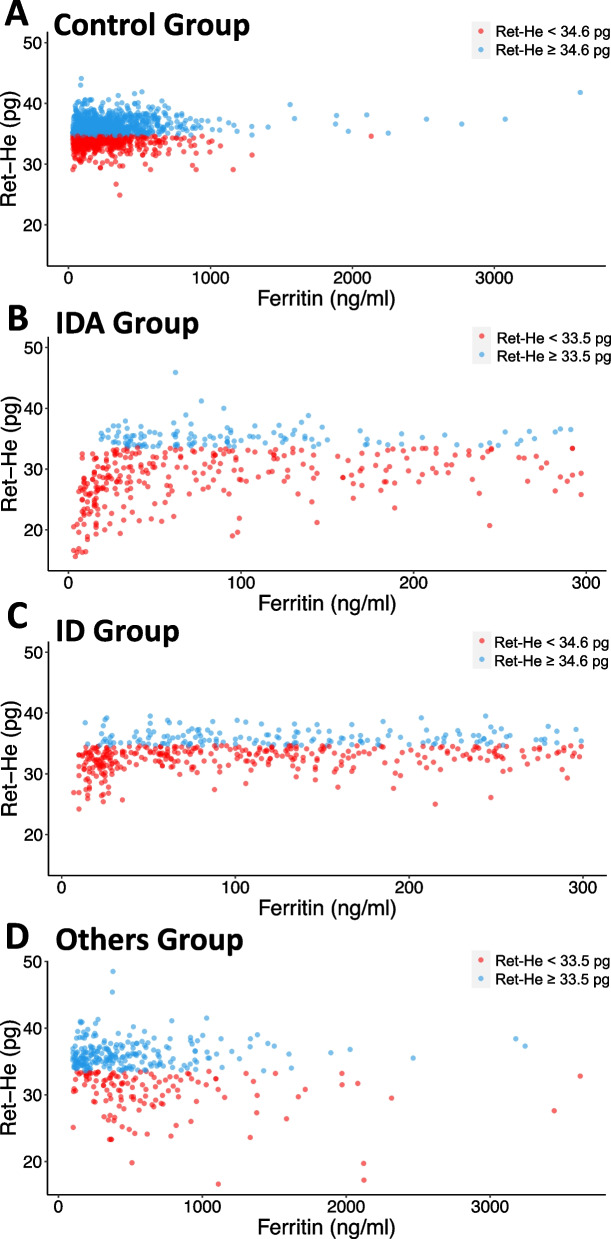
Distribution of Ferritin and Reticulocyte Hemoglobin Content values

The AUC detecting ID was 0.842 (95% CI (0.82–0.87)) in the IDA group, with a sensitivity of 69.7% (95% CI (65.3–74.0%)) and a specificity of 85.7% (95% CI (82.3–86.1%)) at a threshold of 33.5 pg for Ret-He in the IDA group (Fig. 2). Off the 412 patients of the IDA group 303 (73.5%) had Ret-He values < 33.5 pg (Fig. 3B).

The AUC detecting ID was 0.718 (95% CI (0.69–0.75)) in the ID group, with a sensitivity of 65.8% (95% CI (63.3–68.3%)) and a specificity of 67.8% (95% CI (63.2–71.7%)) at a threshold of 34.6 pg for Ret-He in the ID group (Fig. 2). Of the 487 patients of the ID group 319 (65.5%) had Ret-He values < 33.5 pg (Fig. 3C).

In the Control group, 476 (46.5%) patients had Ret-He < 34.6 pg, and in the Other group, 131 (37.4%) patients had Ret-He < 33.5 pg (Fig. 3A and Fig. 3D).

The AUC detecting ID was 0.775 (95% CI (0.76–0.81)) in ID/IDA patients, with a sensitivity of 74.1% (95% CI ((71.2–77.9%)) and a specificity of 65.9% (95% CI ((63.5–68.1%)) at a threshold of 34.6 pg for Ret-He in the ID/IDA patients (Fig. 2).

We performed a classification performance evaluation to assess the binary classification model's effectiveness. For diagnosing ID in anemic patients, a Ret-He cutoff value of 33.5 pg resulted in an accuracy of 62.8%, with a precision of 78.3% and a recall of 72.8%. The F1 Score of 75.4% reflects a balanced trade-off between precision and recall, indicating a reasonable performance despite moderate overall accuracy.

For diagnosing ID in non-anemic patients, using a Ret-He cutoff of 34.6 pg yielded an accuracy of 67.6%, with a precision of 76.1% and a recall of 68.3%. The F1 Score of 72.1% again indicates a balanced trade-off between precision and recall, suggesting that the model performs reasonably well by correctly identifying positive cases while limiting false positives.

The fivefold cross-validation demonstrated an AUC of 0.84 (95% CI (0.80–0.88)) with a specificity of 98% (95% CI (97.1–98.9%)) and a sensitivity of 48.8% (95% CI: 42.9–54.6%) in IDA patients. For ID patients, the AUC was 0.72 (95% CI (0.71–0.75)), with a specificity of 97.6% (95% CI (96.1–99.2%)) and a sensitivity of 19.1% (95% CI (17.3–20.7%)) (Additional Fig. 2).

We also calculated the sensitivity of Ret-He at a specificity greater than 90% for diagnosing ID in both anemic and non-anemic patients. A specificity of over 90% was achieved in both groups using a Ret-He cutoff value of 33.1 pg, with a sensitivity of 63.6% in anemic patients and 35.5% in non-anemic patients.

### Anemia of unknown causes

Off the 370 patients with anemia of unknown cause (Others group) 131 had Ret-He levels < 33.5 pg. In these patients, the median values of laboratory parameters were as follows: ferritin 492.0 ng/ml (333.5; 818.5 ng/ml), transferrin saturation 11.9% (18.0; 23.3%), transferrin receptor 3.9 mg/l (3.1; 5.3 mg/l), MCH 28.5 pg (26.9; 29.5 pg), MCV 85.7 fl (82.5; 89.3 fl), and CRP 2.8 mg/dl (0.4; 7.7 mg/dl) (Additional Fig. 3).

### Predictors for iron deficiency

The logistic regression analysis identified several significant predictors of ID. Lower Hb levels were associated with a 19.4% increase in the odds of ID (OR = 0.806; *p* < 0.001), while a decrease in Ret-He was associated with a 26% increase in odds (OR = 0.740; *p* < 0.001). Additionally, each unit increase in CRP was associated to a 15.1% decrease in the odds of ID (OR = 0.849; *p* < 0.001). In contrast, mean corpuscular volume (MCV), mean corpuscular MCH, and anemia status did not show significant associations with ID (Additional Fig. 4).

**Fig. 4:**
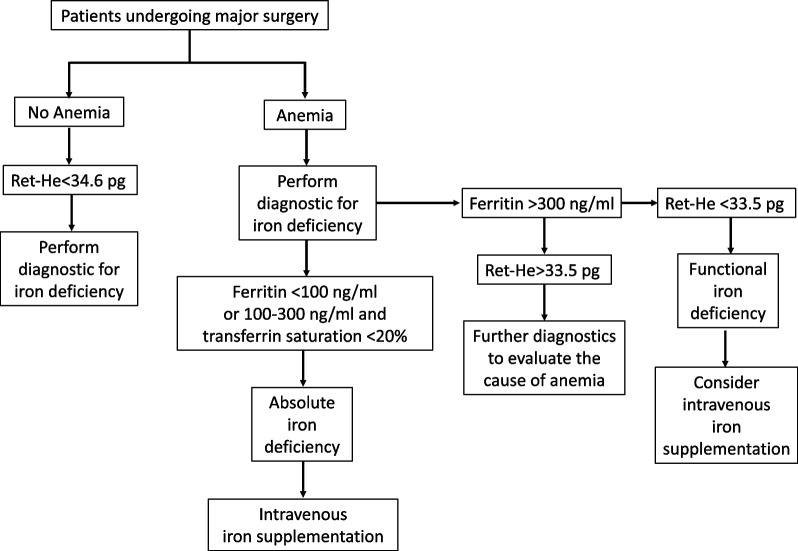
Screening algorithm

## Discussion

Iron deficiency is the leading cause of anemia in both the general population and among patients undergoing major surgery [1]. The importance of screening and treatment of ID before major surgery is increasing. However, performing laboratory analysis for ID is costly. In addition, defining ID can be challenging due to the lack of standardized diagnostic approaches. Various methods are employed, often relying solely on ferritin or using a combination of ferritin, soluble transferrin receptor, transferrin saturation, and CRP. Furthermore, the cutoff values for ferritin levels can vary significantly from 15 ng/ml in healthy individuals to as high as 300 ng/ml in patients with inflammation, chronic kidney disease, or heart failure [5, 6].

In this context, reticulocytes, which are immature red blood cells released from the bone marrow into circulation one to two days before maturation, can provide valuable insights. An increase in the reticulocyte count indicates heightened erythropoiesis. Therefore, the Ret-He may serve as a promising marker that offers a real-time reflection of iron availability for red blood cell production, potentially enhancing the diagnosis of ID [11, 12].

This study evaluated whether Ret-He is a useful marker for predicting ID in 2,769 patients undergoing major surgery. The patients were divided into four groups according to their iron and anemia status. Our results showed that the patients in the IDA group had significant lower Ret-He levels compared to patients of the Control, ID and Others group.

We used ROC analysis to assess the diagnostic performance of Ret-He in defining ID by determining the optimal cutoff value that maximizes both sensitivity (true positive rate) and specificity (true negative rate), thus distinguishing between individuals with and without ID.

A cutoff value of 33.5 pg for Ret-He, the sensitivity was 69.4% and specificity was 85.7%, corresponding to an AUC of 0.842 in the IDA group. This means that 30.6% of the anemic patients with ID would not be correctly diagnosed as iron deficient. To validate our observation, a classification model was performed. The model showed that a cutoff value of 33.5 pg for Ret-He in anemic patients is effective at identifying positive cases (as indicated by the high precision and recall). However, the overall accuracy (62.8%) is moderate, suggesting that using a cutoff of 33.5 pg may miss or misclassify some patients, especially in borderline cases. It is noteworthy that the 30.6% of anemic patients who were not correctly diagnosed likely represent cases where Ret-He and ferritin differ in their ability to detect ID due to biological variability or mixed anemia types. Anemia can have multiple causes beyond ID, including chronic inflammation, vitamin deficiencies, chronic kidney disease, and bone marrow disorders. In these cases, anemia may be present, but ID may not be the primary driver. This could explain the observed weak correlation between ferritin and Ret-He.

Several studies assessed the efficiency of Ret-He in diagnosing ID [7, 13–17]. The suggested cutoff values for Ret-He range from 25.7 pg with an AUC of 0.931 (71.4% sensitivity and 100% specificity) [18], 28.5 pg with an AUC of 0.902 (68% sensitivity and 90% specificity) [7], 32.4 pg with an AUC of 0.753 (72.5% sensitivity and 70% specificity) [19], to 35.5 pg with an AUC of 0.881 (100% sensitivity and 55.6% specificity) [18]. This variability is likely due to the use of different ferritin thresholds to diagnose ID. A higher ferritin cutoff might lead to capturing more mild cases of ID, which can cause the Ret-He cutoff to shift to maintain a balance between correctly identifying iron deficiency (sensitivity) and avoiding false positives (specificity).

Three hundred seventy patients had anemia but no ID according to the definition we used in this study. Of these, 131 patients had Ret-He levels below 33.5 pg. The combination of low transferrin saturation, high ferritin, normal transferrin receptor levels, and elevated CRP in these patients suggests anemia of chronic disease rather than classic IDA. Anemia of chronic disease is common in conditions characterized by chronic inflammation, such as infections, autoimmune diseases, or malignancies, where iron metabolism is disrupted due to the inflammatory response. In these cases, iron becomes sequestered in storage sites, leading to reduced availability for red blood cell production, despite overall iron stores being sufficient or even elevated. The normal MCV and MCH further support the diagnosis of anemia of chronic disease. Additionally, there may be some degree of functional ID, where iron is present but not readily available for use by the bone marrow [7, 13–15]. This may explain the low Ret-He and transferrin saturation, despite adequate or elevated ferritin levels.

Chinudomwong et al. analyzed 953 individuals and suggested that a Ret-He cutoff > 30 pg could potentially be used to exclude IDA. However, the authors state that in the context of diagnosing ID, the evaluation of serum ferritin remains necessary [20]. The fivefold cross-validation showed an AUC of 0.842 in IDA patients. The high sensitivity (98%) with lower specificity (48.8%) suggests that the Ret-He cutoff of 33.5 pg is particularly good at identifying ID but may overestimate the number of patients who are iron deficient.

Several potential confounding factors could impact the accuracy and interpretation of Ret-He during the diagnosis of ID, as factors beyond iron availability may influence Ret-He level [21–27]. Ret-He reflects the amount of hemoglobin incorporated into reticulocytes during their development in the bone marrow. Disruptions in this process can arise from conditions affecting erythropoiesis, Hb synthesis, or red blood cell maturation. For instance, deficiencies in vitamins such as B12, B9 (folate), or A can reduce Ret-He levels by impairing Hb production or erythroid cell development. Additionally, bone marrow function can be altered by factors like chemotherapy, radiation therapy, or myelodysplastic syndromes, all of which may reduce reticulocyte production and, consequently, Ret-He. Hormonal imbalances, such as insufficient erythropoietin or thyroid hormone levels, can also impair reticulocyte production. Furthermore, genetic disorders, viral infections (e.g., Plasmodium or Parvovirus B19), drug abuse, or the effects of certain medications may affect reticulocyte maturation and hemoglobin incorporation, leading to altered Ret-He levels. It would be interesting to assess these confounding factors in major surgical patients to determine their impact on the diagnostic accuracy of Ret-He for identifying ID. Additionally, evaluating whether tailored approaches are needed to account for these variables in this specific population could provide further insights.

Implementing anemia screening for major surgical patients is challenging due to logistical issues, such as integrating it into preoperative workflows and coordination between medical disciplines, especially with limited time before surgery. Resource constraints, such as limited staffing and financial challenges, exacerbate the difficulty. This is further compounded by the absence of standardized protocols and insufficient clinician awareness. Therefore, a straightforward screening algorithm paired with clear management protocols could help to identify and address anemia efficiently, ensuring timely intervention and improved outcomes for surgical patients.

Overall, the results of the regression analysis underscore Hb and Ret-He as significant predictors of IDA, highlighting their importance in evaluating and managing at-risk patients for this condition. Given the strong association with the odds of developing IDA, this marker can help prioritize patients for ID screening before major surgery using a two-factor scoring tool, as suggested in Fig. 4: Patients undergoing major surgery are first evaluated for the presence of anemia. In patients without anemia, a Ret-He value of < 34.6 pg may indicate ID, and iron diagnostic tests should be performed to confirm this condition. For patients with anemia, iron diagnostics should be conducted as part of the evaluation. If serum ferritin levels are > 300 ng/ml, a Ret-He value of < 33.5 pg may help distinguish functional ID, where iron is sequestered despite adequate stores, from other causes of anemia. If functional ID is ruled out, further diagnostic investigations should be carried out to determine the underlying cause of anemia. It is noteworthy that this retrospective study investigated the significance of Ret-He in diagnosis of ID in major surgical patients. Due to its retrospective design, our study could not evaluate the effectiveness of the workflow suggested in Fig. 4. Future studies could investigate predictive models that incorporate Ret-He for preoperative anemia management.

The diagnosis and treatment of ID in non-anemic patients remains controversial. Although iron supplementation is known to be beneficial in anemic patients, evidence is mixed for non-anemic patients. Some studies show that iron supplementation in these patients can improve postoperative outcome while others show minimal or even no improvement [28–31]. By incorporating Ret-He into anemia management, clinicians can better prioritize patients for further iron parameter assessments, reducing unnecessary testing and healthcare costs in both anemic and non-anemic patients (Fig. 4).

## Conclusion

In conclusion, this study highlights the potential of Ret-He as a promising marker for diagnosing ID in patients undergoing major surgery, particularly in cases of elevated ferritin levels or non-anemic patients. Ret-He can serve as a valuable tool to prioritize patients for further diagnostic testing, especially when preoperative time is limited. Further research is warranted to refine the use of Ret-He and establish more standardized diagnostic thresholds for ID across different clinical contexts.

## Supplementary Information

Supplementary Material 1.

## Data Availability

The datasets used and/or analyzed during the current study are available from the corresponding author on reasonable request.

## Abbreviations

AUC: Area under curve
CRP: C-reactive protein
Hb: Hemoglobin
IQR: Inter quartile range
ID: Iron deficiency
IDA: Iron deficiency anemia
MCH: Mean corpuscular hemoglobin
MCV: Mean corpuscular volume
OR: Odds Ratio
ROC: Receiver Operating Characteristic
Ret-He: Reticulocyte hemoglobin content
WHO: World Health Organization
