# Supplementary material for: Reticulocyte hemoglobin content: a new frontier in iron deficiency diagnostics for major surgical patients
Source: BMC Anesthesiol. 2025 Jan 25;25:40. doi: 10.1186/s12871-025-02905-6 (PMC11762468; doi:10.1186/s12871-025-02905-6)
Supplement: Supplementary file 1 — Supplementary Material 1. [file 12871_2025_2905_MOESM1_ESM.docx]

# Supplemental Table 1: Summary of Spearman's rank correlation (rho):

|  | **Ret-He vs. Ferritin** | **Ret-He vs. Transferrin saturation** |
| --- | --- | --- |
| All | 0.264* | 0.543* |
| Control | 0.107* | 0.289* |
| IDA | 0.370* | 0.641* |
| ID | 0.274* | 0.350* |
| Others | -0.166* | 0.544* |

**Categories of correlation based on rho: 0.00 to 0.39** (**weak); 0.40 to 0.59** (**moderate); 0.60 to 1.00 (strong)**; *statistically significant; ID=Iron deficiency; IDA=Iron deficiency anemia; Ret-He=Reticulocyte Hemoglobin Content

# Supplemental Figure 1: Spearman's rank correlation

ID=Iron deficiency; IDA=Iron deficiency anemia; Ret-He=Reticulocyte Hemoglobin Content

# Supplemental Figure 2: Receiver-operating characteristic analysis of Ret-He using a 5-fold validation approach

# Supplemental Figure 3: Distribution of laboratory parameters

Hb=hemoglobin; MCV=mean corpuscular volume; MCH=mean corpuscular hemoglobin, Ret-He=Reticulocyte hemoglobin content; CRP=C-reactive protein; Tsat=Transferrin saturation

# Supplemental Figure 4: Odds Ratios for Predictors of Iron deficiency

Hb=hemoglobin; MCV=mean corpuscular volume; MCH=mean corpuscular hemoglobin, Ret-He=Reticulocyte hemoglobin content; CRP=C-reactive protein
